# EGFR or HER2 inhibition modulates the tumor microenvironment by suppression of PD-L1 and cytokines release

**DOI:** 10.18632/oncotarget.19194

**Published:** 2017-07-12

**Authors:** Koung Jin Suh, Ji Hea Sung, Jin Won Kim, Song-Hee Han, Hye Seung Lee, Ahrum Min, Mi Hyun Kang, Ji Eun Kim, Ji-Won Kim, Se Hyun Kim, Jeong-Ok Lee, Yu Jung Kim, Keun-Wook Lee, Jee Hyun Kim, Soo-Mee Bang, Seock-Ah Im, Jong Seok Lee

**Affiliations:** ^1^ Department of Internal Medicine, Seoul National University Bundang Hospital, Seoul National University College of Medicine, Bundang-gu, Seongnam, Korea; ^2^ Department of Pathology, Seoul National University Bundang Hospital, Seoul National University College of Medicine, Bundang-gu, Seongnam, Korea; ^3^ Department of Internal Medicine, Seoul National University Hospital, Seoul National University College of Medicine, Jongno-gu, Seoul, Korea; ^4^ Cancer Research Institute, Seoul National University, Jongno-gu, Seoul, Korea

**Keywords:** PD-L1, cytokine, EGFR, HER2, PI3K

## Abstract

**Background:**

Characteristics of tumor microenvironment have been suggested as predictive markers of anti-EGFR or anti-HER2 treatment response. However, the effect of EGFR/HER2 signal blockade on the tumor immune microenvironment is unclear.

**Methods:**

EGFR/HER2 pathway signaling and PD-L1 expression in gastric cancer cell lines were screened by western blot analysis. PD-L1 and HER2 expressions in 251 resected gastric tumors were determined by immunohistochemistry, and changes in EFGR, HER2, and PD-L1 expression in paired specimens between pre- and post-chemotherapy were evaluated. PD-L1 expression in HER2-amplified cell lines was evaluated by western blotting, fluorescence-activated cell sorting, reverse transcription, and real-time quantitative PCR analyses before and after afatinib, lapatinib, pictilisib and trametinib treatment. Changes in cytokines were evaluated by reverse transcription, real-time quantitative PCR, and enzyme-linked immunosorbent assay after EGFR/HER2 inhibition.

**Results:**

Cell lines with pEGFR or pHER2 overexpression showed higher PD-L1 expression. In resected gastric tumors, HER2 expression was significantly associated with PD-L1 expression (*p*=0.030). PD-L1 overexpression accompanied by increased HER2 expression was identified in a post-chemotherapy specimen from a patient with an initial HER2/PD-L1-negative tumor. In HER2-overexpressing cell lines, PD-L1 expression was decreased in a dose- and time-dependent manner after afatinib and lapatinib treatment. PI3K pathway inhibition by pictilisib, but not MEK pathway inhibition by trametinib, resulted in PD-L1 suppression. After lapatinib treatment, the release of CCL2, CCL21, VEGF and CXCL1 decreased in a dose-dependent manner.

**Conclusions:**

Inhibition of the EGFR/HER2 signaling pathway, particularly of downstream PI3K activity, suppressed PD-L1 and release of cytokines, suggesting that EGFR/HER2 inhibition may create a more favorable milieu for tumor immunotherapy.

## INTRODUCTION

The interaction between tumors and the tumor microenvironment is complicated. Tumor cells and the components of the tumor microenvironment communicate directly through cell-to-cell contact and indirectly through paracrine signals, which predominantly involve cytokines [1, 2]. The programmed cell death-ligand 1 (PD-L1) on tumor cells has an important role in avoiding host immune surveillance by interacting with the programmed cell death-1 (PD-1) on immune cells in the tumor microenvironment [3, 4]. Cytokines that are secreted by the tumor cells or by the normal cells recruited to the tumor microenvironment can function as growth factors, increase metastasis formation and angiogenesis, and induce the formation of an immunosuppressive microenvironment [5, 6].

Targeting the PD-1/PD-L1 immune checkpoint signaling to restore cancer cell-directed immune response is a confirmed strategy for several tumor types. Although there are controversies, PD-L1 expression has been suggested as a predictive marker of PD-1/PD-L1 checkpoint inhibitor treatment response [7–9]. Recent studies have suggested that the predictive value of PD-L1 overexpression might not be restricted to immune checkpoint inhibitor treatments. D'Incecco et al. reported that in patients with non-small cell lung cancer (NSCLC) treated with epidermal growth factor receptor (EGFR) tyrosine kinase inhibitors (TKIs), the response rate, time to progression, and overall survival (OS) were significantly better in PD-L1-positive tumors [10]. High PD-L1 expression was positively associated with EGFR mutation status [11] and better EGFR TKI treatment outcome in patients with EGFR-mutant NSCLC [12]. In addition, higher levels of tumor-infiltrating lymphocytes (TILs), another important participant in antitumor immunity, were significantly associated with decreased distant recurrence in patients with HER2-positive breast cancer treated with the anti-HER2 monoclonal antibody, trastuzumab [13]. Taken together, these data indicate that overexpression of PD-L1 is associated with EGFR/HER2 status, and drugs targeting these pathways may relieve suppression of antitumor effector immunity. However, the mechanism by which oncogenic signaling modulates tumor immunity in the tumor microenvironment has not been elucidated. There are only a few studies that have addressed this issue by investigating the oncogenic activation of the AKT-mTOR pathway [14, 15] or the MEK-ERK pathway [15] and NF-Kb regulation [16].

To elucidate the effect of EGFR/HER2 signal blockade on the tumor immune microenvironment, we examined the association between PD-L1 expression and the EGFR/HER2 signaling pathway and the direct effect of EGFR/HER2 pathway inhibition on the cytokines release by tumor cells.

## RESULTS

### Association between PD-L1 and EGFR/HER2 signaling in gastric cancer cell lines

**Figure 1:**
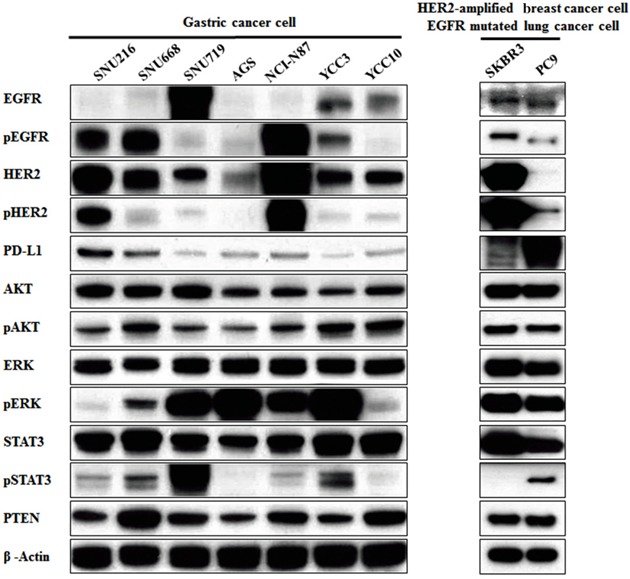
Association of PD-L1 expression with the EGFR/HER2 signaling pathway in gastric cancer cell lines Seven gastric cancer cell lines (SNU216, SNU668, SNU719, AGS, NCI-N87, YCC3 and YCC10) were seeded at a density of 1 × 10^6^ cells per well in 6-well plates and incubated in a humidified CO_2_ incubator at 37°C. The protein expressions of molecules related to the EGFR/HER2 signaling pathway were screened by western blot analysis. PD-L1 expression in cell lines with pEGFR overexpression or pHER2 overexpression (SNU216, SNU668, and NCI-N87) was relatively higher than that in other cell lines. As positive controls, HER2-amplified breast cancer cell line (SKBR3) and EGFR-mutated lung cancer cell line (PC9) showed higher PD-L1 expression.

To determine the association between PD-L1 and the EGFR/HER2 pathway, we examined the protein expressions of PD-L1 and molecules related to the EGFR/HER2 signaling pathway in seven gastric cancer cell lines. Western blot analysis revealed that SNU216, SNU668, and NCI-N87 had higher p-EGFR, p-HER2, and PD-L1 expression (Figure 1). These results suggest a positive association between PD-L1 expression and EGFR/HER2 signaling pathway activation. This finding was also confirmed in HER2-amplified breast cancer cell line (SKBR3) and EGFR-mutated lung cancer cell line (PC9) as positive controls.

To determine the clinical relevance of the findings, we evaluated PD-L1 and HER2 expressions in 251 surgically resected gastric tumor tissues from our previously reported gastric cancer cohort [17]. PD-L1 and HER2 protein expressions (1+, 2+, 3+) in 173 (68.9%) and 68 (27.1%) patients, respectively, were evaluated. Among the patients with negative HER2 protein expression (n = 183), 63.4% had positive PD-L1 expression (n = 116). PD-L1 expression was more frequent in patients with positive HER2 protein expression; 89.5% (34/38), 75.0% (12/16), and 78.6% (11/14) of the patients with 1+, 2+, and 3+ HER2 expression, respectively, had PD-L1-positive tumors, thus demonstrating a significant linear association (*p* = 0.030) (Table 1).

**Table 1:** Association between PD-L1 expression status and clinicopathological characteristics

Variables	PD-L1		Total 251	*P*-value
	Positive (173)	Negative (78)		
HER2 IHC Negative	116 (63.4%)	67 (36.6%)	183	0.030
HER2 IHC 1+	34 (89.5%)	4 (10.5%)	38	
HER2 IHC 2+	12 (75.0%)	4 (25.0%)	16	
HER2 IHC 3+	11 (78.6%)	3 (21.4%)	14	

IHC immunohistochemistry. HER2 immunohistochemistry results are scored as IHC 0 (negative), IHC 1+ (negative), IHC 2 + (equivocal) or IHC 3+ (positive).

**Figure 2:**
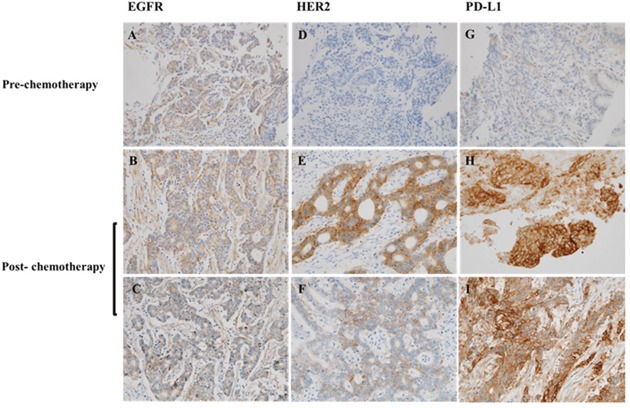
Immunohistochemistry analysis of the paired tumor specimens of pre-chemotherapy and post-chemotherapy The specimens were stained for **(A-C)** EGFR, **(D-F)** HER2, and **(G-I)** PD-L1 expression. HER2 and PD-L1 were overexpressed in the post-chemotherapy specimen of an initial HER2/PD-L1-negative tumor; original magnification ×400.

The EGFR, HER2, and PD-L1 expressions in paired specimens of tumors obtained pre- and post-chemotherapy were evaluated by immunohistochemistry (IHC). A total of 10 specimens from five patients were included in this analysis. In one patient with an initial HER2-negative tumor, the post-chemotherapy specimen showed increased PD-L1 and HER2 expression levels (Figure 2). In the other four patients, the expression levels of EGFR, HER2, and PD-L1 did not show any difference between pre- and post-chemotherapy.

### Changes in PD-L1 expression following EGFR/HER2 signaling blockade in HER2-amplified cell lines

**Figure 3:**
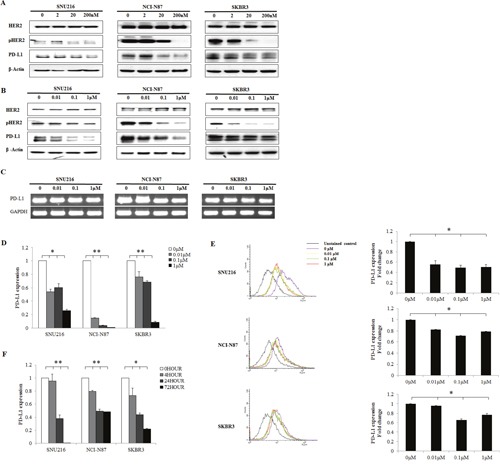
Decrease in PD-L1 expression following EGFR/HER2 inhibition in HER2-amplified cell lines HER2-amplified gastric (SNU216, NCI-N87) and breast (SKBR3) cancer cell lines were seeded at a density of 1 × 10^6^ cells per well in 6-well plates and incubated in a humidified CO_2_ incubator at 37°C. After overnight growth, **(A)** afatinib (0, 2, 20, and 200 nM) and **(B)** lapatinib (0, 0.01, 0.1, and 1 μM) were added. Cells were harvested at 24 h, and the expression of PD-L1 was analyzed by western blot analysis. PD-L1 was suppressed in a dose-dependent manner. The mRNA expression of PD-L1 after lapatinib (0, 0.01, 0.1, and 1 μM) treatment was determined by **(C)** RT-PCR and **(D)** qPCR. **(E)** The mean fluorescence intensity of PD-L1 was determined by flow cytometry after lapatinib (0, 0.01, 0.1, and 1 μM) treatment. **(F)** PD-L1 expression was examined by qPCR after treatment with 0.1 μM lapatinib for different times (0, 4, 24, and 72 h). The PD-L1 expression of three independent experiments is shown as mean ± SD. *0.001 ≤ *p* < 0.05 and **p < 0.001 in ANOVA.

To determine the effect of EGFR/HER2 signaling pathway inhibition on PD-L1 expression, the changes in PD-L1 expression following EGFR/HER2 blockade in HER2-amplified cell lines (SNU216, NCI-N87, and SKBR3) were evaluated using afatinib and lapatinib (dual kinase inhibitor of EGFR and HER2). The protein expression of PD-L1 was suppressed in a dose-dependent manner when pHER2 was suppressed by lapatinib and afatinib (Figure 3A and 3B). The mRNA expression of PD-L1 was also reduced in a dose-dependent manner, as determined by reverse transcription PCR (RT-PCR) and real-time quantitative PCR (qPCR) (Figure 3C and 3D). The expression of PD-L1 was decreased with increasing lapatinib treatment, as assessed by flow cytometer (Figure 3E). Moreover, when the cell lines were treated with 0.1 μM lapatinib for different times (0, 4, 24, and 72 h), a gradual pattern of PD-L1 suppression was observed (Figure 3F). These data indicate that EGFR/HER2 blockade could reduce the expression of PD-L1 in HER2-amplified cancer cells.

### PD-L1 suppression via inhibition of AKT signaling in HER2-amplified cell lines

**Figure 4:**
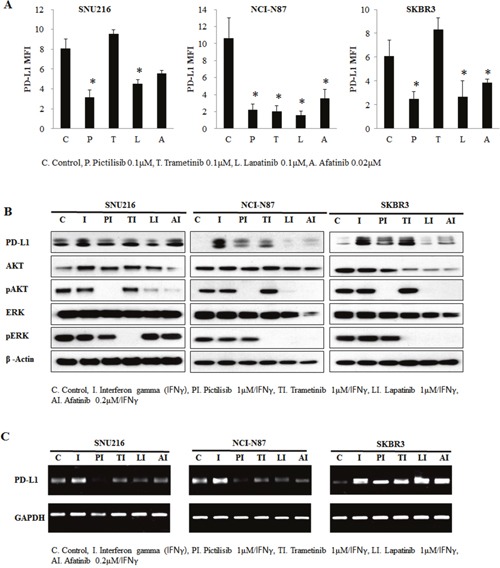
Involvement of the AKT pathway in PD-L1 expression regulation HER2-amplified cancer cell lines were seeded at a density of 1 × 10^6^ cells per well in 6-well plates and incubated in a humidified CO_2_ incubator at 37°C. After overnight growth, cells were treated with 0.1 μM pictilisib, 0.1 μM trametinib, 0.1 μM lapatinib, and 0.02 μM afatinib for 24 h. **(A)** The mean fluorescence intensity (MFI) of PD-L1 was determined by flow cytometry. PD-L1 was expression decreased in pictilisib-treated cell lines. In contrast, PD-L1 expression was not suppressed in trametinib-treated SNU216 and SKBR3 cell lines. To enhance PD-L1 expression, IFNγ (10 ng/mL) was added after 4 h of drug treatment, and incubation was continued for a further 24 h. At 28 h after drug treatment, cells were harvested for evaluation of PD-L1 protein expression by **(B)** western blotting and **(C)** RT-PCR analyses. In comparison with trametinib inhibition, pictilisib inhibition of the AKT pathway suppressed IFNγ-mediated PD-L1 protein upregulation. The PD-L1 expression of three independent experiments is shown as mean ± SD. *p < 0.05 in paired Student's t-test (2-tailed).

The PI3K-AKT-mTOR and RAS-RAF-MEK pathways are two of the most important downstream pathways of EGFR/HER2 signaling. We hypothesized that one of these downstream pathways could be dominantly involved in the regulation of PD-L1 by HER2 overexpression. To determine whether PD-L1 expression was dependent on active PI3K-AKT-mTOR signaling or RAS-RAF-MEK signaling, HER2-amplified cell lines with high PD-L1 expression were treated with pharmacologic inhibitors of specific components in the pathway. We treated the SNU216, NCI-N87, and SKBR3 cell lines with 0.1 μM pictilisib (a selective inhibitor of class I PI3Ks), 0.1 μM trametinib (a MEK inhibitor), 0.1 μM lapatinib, and 0.02 μM afatinib. By using flow cytometer, we found that the expression of PD-L1 was reduced in pictilisib-treated, lapatinib-treated, and afatinib-treated cell lines (Figure 4A). In contrast, the expression of PD-L1 was not suppressed in the trametinib-treated SNU216 and SKBR3 cell lines.

To more clearly demonstrate the effect of inhibition, cell lines were treated with interferon gamma (IFNγ) to induce PD-L1 expression. Indeed, PD-L1 expression in the cell lines was stimulated after IFNγ treatment. Pictilisib but not trametinib inhibited the induction of PD-L1 through IFNγ both at the protein and mRNA level (Figure 4B and 4C). These results suggest the association of ERGF/HER2 with PD-L1 via the PI3K-AKT-mTOR pathway dominantly.

### Changes in cytokines release following EGFR/HER2 blockade

**Figure 5:**
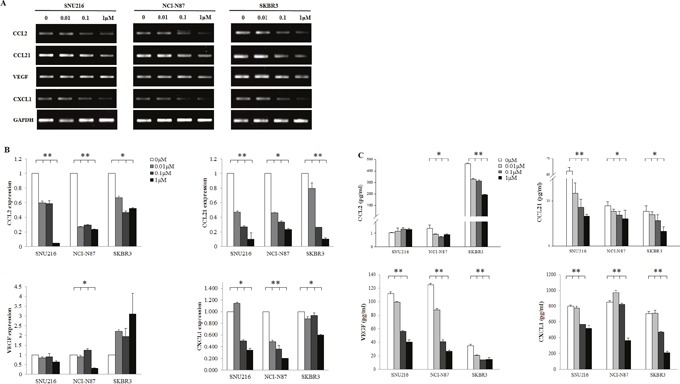
Effect of HER2 blockade on the cytokine release of tumor cells HER2-amplified cell lines were seeded at a density of 1 × 10^6^ cells per well in 6-well plates and incubated in a humidified CO_2_ incubator at 37°C. After overnight growth, lapatinib (0, 0.01, 0.1, and 1 μM) was added. Cells were harvested after 24 h. The mRNA expression of CCL2, CCL21, VEGF, and CXCL1 was examined by **(A)** RT-PCR, **(B)** qPCR, and **(C)** ELISA which demonstrated a dose-dependent suppression. The expression of three independent experiments is shown as mean ± SD. *0.001 ≤ *p* < 0.05 and **p < 0.001 in ANOVA.

We examined the direct effect of EGFR/HER2 inhibition on the cytokines release of tumor cell lines. SNU216, NCI-N87, and SKBR3 cell lines were treated with different concentrations of lapatinib (0, 0.01, 0.1, and 1 μM). The mRNA expression of CCL2, CCL21, and CXCL1 was suppressed in a dose-dependent manner (Figure 5A and 5B). Furthermore, in enzyme-linked immunosorbent assay (ELISA) of supernatants after 24h treatment with lapatinib, the concentration of CCL2, CCL21, VEGF and CXCL1 were decreased in a dose-dependent manner, although dramatic decrease of CCL2 concentration did not be shown due to very low baseline concentration in SNU216 and NCI-N87.

## DISCUSSION

In the present study, we demonstrated that PD-L1 expression was higher in gastric cancer cell lines with higher pEGFR and pHER2 expressions compared with those with lower pEGFR and pHER2 expressions, and inhibition of the HER2/EGFR pathway decreased PD-L1 expression as well as cytokines release in HER2-amplified cancer cell lines. Furthermore, inhibition of the PI3K but not MEK pathway reduced PD-L1 expression in these cell lines, thus suggesting that the modulation of PD-L1 expression by the EGFR/HER2 pathway is likely to be mediated via downstream PI3K-AKT-mTOR signaling.

The interaction between tumors and the tumor microenvironment is complicated. Signaling pathways of the inner tumor can be associated with the tumor microenvironment of the outer tumor. The tumor microenvironment also influences tumor behavior. PD-L1 is a transmembrane surface glycoprotein expressed by lymphocytes and dendritic cells as well as aberrantly on the surface of epithelial cells in a wide range of solid tumors. In addition, PD-L1 is a ligand of PD-1, which is known to be expressed by activated T-cells in the germinal centers of lymphoid follicles as well as TILs [3]. The interaction between PD-1 and its ligand PD-L1 suppresses T-cell receptor signaling and results in immune system downregulation, which in turn enables cancer cells to escape immune destruction [4]. Several cytokines, such as CCL2, CCL21, VEGF and CXCL1 are present at high levels in the tumor microenvironment and known to be involved in tumor growth promotion, vascular development, and evasion of antitumor immunity. These molecules are thought necessary for tumors to interact with their microenvironment. In this study, decrease of cytokines release after lapatinib treatment was reported, which might give an indirect evidence for tumor treatment itself to modulate tumor microenvironment.

Recent studies have demonstrated that PD-L1 expression is not only a marker of response to immune checkpoint inhibitor treatment but also cytotoxic chemotherapy and targeted therapy. PD-L1 expression can predict pathologic complete response in neoadjuvant chemotherapy-treated patients with breast cancer [19]. A study in patients with EGFR-mutant NSCLC following EGFR TKI therapy revealed that the outcome of patients with PD-L1-positive tumors was better compared with those with PD-L1-negative tumors [20]. Furthermore, another study found significantly greater levels of PD-L1 through EGFR pathway activation after acquiring resistance to gefitinib in NSCLC cell lines [21]. An increase in PD-L1-positive peripheral blood T-cells after EGFR TKI treatment has been strongly associated with disease progression, thus suggesting that PD-L1 upregulation might also be one of the mechanisms of acquired resistance to EGFR TKIs [22]. Therefore, we suggest that overexpression of PD-L1 is associated with the EGFR/HER2 pathway, and the drugs targeting these pathways may relieve suppression of antitumor effector immunity. Since prognosis and treatment response of agents targeting the growth signaling pathways of the inner tumor are different depending on the tumor microenvironment, it is important to evaluate the effect of tumor oncogenic signaling on the tumor microenvironment. We found that the PI3K pathway was associated with PD-L1 expression. Similar to the present study, Lastwika et al. recently reported a strong association between PD-L1 protein expression and activation of the AKT-mTOR pathway in lung cancer [14]. They found that inhibition of PI3K, AKT, or mTOR decreased PD-L1 expression in EGFR-mutated NSCLC cell lines, which was similar to our finding in HER2-amplified cancer cell lines. Ota et al. also reported that expression of PD-L1 was suppressed by inhibitors of the MEK–ERK and PI3K–AKT signaling pathways in NSCLC cells positive for either EML4–ALK or activating mutations of the EGFR [15]. These reports including our result reveal an indirect link between tumor oncogenic signaling and tumor microenvironment.

In our tissue microarray data, HER2 was expressed in approximately 33% of tumors with PD-L1 expression, and PD-L1 was expressed in 84% of tumors with HER2 protein expression. HER2 amplification was not associated with PD-L1 expression in our cohort, which is in contrast with a study reporting HER2 overexpression in nearly 50% of PD-L1-positive gastric cancer cases [23]. According to TCGA data, which divided gastric cancer into four distinct subtypes [24], PD-L1/L2 expression is elevated in EBV-positive tumors; in contrast, HER2 amplification is most frequently found in chromosomal instability subtypes. These data along with our findings suggest that PD-L1 status is not always associated with HER2 amplification. However, one paired specimen demonstrated increased PD-L1 and HER2 expression after chemotherapy in the present study. Although PD-L1 status might not be associated with HER2 expression, PD-L1 expression could be activated or suppressed by the EGFR/HER2 pathway.

Our study has several limitations. The effect of EGFR/HER2 inhibition on PD-L1 and cytokine release was only evaluated in pEGFR and/or pHER2 overexpressed cell lines; thus, a possible association between the EGFR/HER2 pathway and PD-L1 expression in other cell lines could have been overlooked. To address the microenvironment impact of the treatment, 2-dimensional experiments have limitation. Although co-culture system with immune cells may be better to prove the concepts, this study could not incorporate this system. In addition, we were unable to evaluate the change in PD-L1 expression and cytokines release in tumor tissues obtained from patients treated with different inhibitors used in this study (PI3K inhibitor and MEK inhibitor) since these agents are not used in routine clinical practice. Nevertheless, paired tissues of pre-chemotherapy and post-chemotherapy were available and the association between PD-L1 and EGFR/HER2 was evaluated. Further *in vivo* studies are warranted to confirm the findings in our *in vitro* study.

Overall, our findings suggest that agents targeting EGFR/HER2 could inhibit the induction of PD-L1, which may be dependent on the PI3K-AKT-mTOR pathway and suppress cytokines release in HER2-amplified cancer cells. EGFR/HER2 inhibition may create a more favorable milieu for tumor immunotherapy.

## MATERIALS AND METHODS

### Cell lines and reagents

NCI-N87, AGS, and SKBR3 cells were obtained from the American Type Culture Collection. SNU216, SNU668, and SNU719 cells were obtained from the Korean Cell Line Bank. YCC3, YCC10, and PC9 cells were obtained from Cancer Metastasis Research Center of Yonsei University College of Medicine. All cell lines were authenticated using by short tandem repeats. All were cultured in RPMI 1640 (Gibco, grand Island, NY) with L-glutamine (300 mg/L), supplemented with 25 mM HEPES, 25 mM NAHCO_3_, 10% fetal bovine serum (FBS; Gibco, grand Island, NY), penicillin (100 U/mL), and streptomycin (50 μg/mL, WelGENE Inc, Daegu, Korea). The cell cultures were maintained in a humidified CO_2_ incubator at 37°C. The drugs including Afatinib, pictilisib, trametinib, and lapatinib were purchased from Selleck Chemicals (Houston, TX, USA). IFNγ was obtained from ProSpec-Tany TechnoGene Ltd. (Rehovot, Israe). SNU216, NCI-N87, and SKBR3 were seeded in 6-well tissue culture plates at a density of 1 × 10^6^ cells per well. Cells were cultured in RPMI 1640 with 10% fetal bovine serum. After overnight growth, the cells were treated with specific drugs at different concentrations and time points. After each exposure time of drugs, cells were harvested for experiments. To enhance PD-L1 expression, IFNγ-induction was applied. After cell seeding and overnight growth, the cells were treated with specific drugs. And then, IFNγ (10 ng/mL) was added after 4h of drug treatment, and incubation was continued for a further 24h. At 28 h after drug treatment, cells were harvested for experiments.

### Western blot analysis

Cells were lysed in 1× lysis buffer (Cell Signaling Technology, Danvers, MA, USA) containing 1 mM of the protease inhibitor PMSF (Amresco, Solon, OH, USA), and the cell extracts were centrifuged at 16000g for 15 min. All samples were denatured in buffer containing 60 mM Tris, pH 6.8, 25% glycerol, 2% SDS, 14.4 mM 2-mercaptoethanol, and 0.1% bromophenol blue at 100°C for 5 min. SDS polyacrylamide gels (Bio-Rad laboratories, Hercules, CA, USA) were loaded with 25 μg total protein per lane. Prestained protein molecular weight markers (Bio-Rad) were run as standards. The electrophoresed samples were transferred to PVDF membranes (Millipore, Billerica, MA, USA). After transfer, the membrane was blocked with 5% skim milk in TPBS (200 mM Tris pH 7.0, 1.37 M NaCl, 1% Tween-20) for 1 h at room temperature. The anti-PD-L1 was obtained from Abcam (Cambridge, MA, USA). The antibodies including anti-HER2, anti-phospho-HER2, anti-Akt, anti-phospho-Akt, anti-ERK, anti-phospho-ERK, anti-STAT3, anti-phospho-STAT3, anti-PTEN, and anti-beta actin antibodies were purchased from Cell Signaling Technology (Danvers, MA, USA). The membrane was then incubated with diluted primary antibodys for overnight at 4°C, rinsed and washed, and incubated with the appropriate HRP-conjugated secondary antibody (1:4,000 dilution; Cell Signaling Technology, Danvers, MA, USA) for 2h at room temperature. The membrane was washed and rinsed as before, and the expressed proteins were detected with the Pierce ECL Plus Western Blotting Substrate (Amersham Pharmacia Biotech, Buckinghamshire, UK). Each experiment was performed in triplicate.

### Flow cytometry analysis

Cells were centrifuged at 300 g for 3 min, washed with PBS, and fixed in 70% ethanol at −20°C. After incubation with fluorescently labeled antibodies for 30 min at 4°C, cells were washed with PBS buffer and analyzed on a FACSCalibur flow cytometer (Becton Dickinson Biosciences, Heidelberg, Germany) with CellQuest Pro software (10000 events were recorded for each sample).

### qPCR and RT-PCR

Total mRNA was purified from cells using TRIzol reagent (Invitrogen, carsbad, CA, USA) according to the manufacturer's protocol for cultured epithelial cells. The High Capacity RNA-to-cDNA Kit (Applied Biosystems, CA, USA) was used to generate cDNA from 1 μg of total mRNA. qPCR analysis was performed on the ABI qPCR system (Applied Biosystems) using SYBR Green PCR Master Mix (Applied Biosystems). The RT-PCR amplification program used was 5 min at 94°C followed by 35 cycles at 94°C for 30 s, 55°C for 30 s, and 72°C for 30 s. The PCR products were analyzed on 1% agarose gels (USB Corporation, MA, USA) in 1% Tris-borate-EDTA buffer stained with DYNE Loading STAR (DYNEBIO, SeongNam, Korea), and they were visualized by a UV transilluminator and recorded using a gel photo imaging system.

The primers used in the PCR reaction were as follows: PD-L1 forward primer 5′-ttgggaaatggaggataaga-3′ and reverse primer 5′-ggatgtgccagaggtagttct-3′, CCL2 forward primer 5′- ccccagtcacctgctgttat-3′ and reverse primer 5′-tggaatcctgaacccacttc-3′, CCL21 forward primer 5′-gccttgccacactctttctc-3′ and reverse primer 5′-caaggaagaggtggggtgta-3′, CXCL1 forward primer 5′-gggaattcaccccaagaac-3′ and reverse primer 5′-caccagtgagcttcctcctc-3′, VEGF forward primer 5′-aaggaggagggcagaatcat-3′ and reverse primer 5′-atctgcatggtgatgttgga-3′, and GAPDH forward primer 5′-gcctcaagatcatcagcaatgcct-3′ and reverse primer 5′-tgtggtcatgagtccttccacgat-3′. The relative quantity of mRNA (RQ value) was calculated as RQ = 2^ΔΔCT^.

### ELISA

The changes in release of CCL2, CCL21, VEGF and CXCL1 were evaluated using ELISA. Supernatants were collected after 24h treatment with lapatinib. The experiment was conducted using individual Human ELISA Kits (Abcam, Cambridge, MA, USA) according to the manufacturer's protocols. The absorbance was read at 450 nm using a SPECTRA max PLUS 384(Molecular Devices, CA, USA) and data were analyzed with SoftMax Pro software (Molecular Devices, CA, USA).

### IHC analysis

Tissue microarray construction was performed for 251 patients with gastric cancer who underwent gastric resection between 2003 and 2004 at Seoul National University Bundang Hospital, as previously described [17]. Paired specimens were used to observe changes before or after chemotherapy. Commercially available primary antibodies were used according to the manufacturer's instructions (anti-PD-L1 rabbit polyclonal antibody, 1:1500 dilution [Abcam, Cambridge, MA, USA]; anti-HER2/neu rabbit monoclonal antibody [Ventana, AZ, USA]; anti-EGFR mouse monoclonal antibody [Ventana, AZ, USA]). The IHC staining result for PD-L1 was evaluated and categorized as positive when the intensity of PD-L1 was graded as weak, moderate, or strong in ≥10% of the tumor. HER2 expression was classified as negative, 1+, 2+, or 3+ as a guideline [18].

### Statistical analysis

All the experiments were repeated three times. The values shown in the tables and figures represent the mean ± standard deviation of three assays. Associations among groups were evaluated using chi-square test, linear-by-linear association, and one-way analysis of variance (ANOVA). This study for patients’ information was approved by the Institutional Review Board of SNUBH (B-1701/378-305). All analyses were performed using Microsoft Excel, SigmaPlot software, and SPSS (SPSS Inc., Chicago, IL, USA).
